# Transfer-learning is a key ingredient to fast deep learning-based 4D liver MRI reconstruction

**DOI:** 10.1038/s41598-023-38073-1

**Published:** 2023-07-11

**Authors:** Gino Gulamhussene, Marko Rak, Oleksii Bashkanov, Fabian Joeres, Jazan Omari, Maciej Pech, Christian Hansen

**Affiliations:** 1 Otto-von-Guericke University Magdeburg, Faculty of Computer Science, 39106 Magdeburg, Germany; 2 Department of Radiology and Nuclear Medicine, University Hospital Magdeburg, 39120 Magdeburg, Germany

**Keywords:** Biomedical engineering, Cancer therapy, Computational science, Computer science, Software

## Abstract

Time-resolved volumetric magnetic resonance imaging (4D MRI) could be used to address organ motion in image-guided interventions like tumor ablation. Current 4D reconstruction techniques are unsuitable for most interventional settings because they are limited to specific breathing phases, lack temporal/spatial resolution, and have long prior acquisitions or reconstruction times. Deep learning-based (DL) 4D MRI approaches promise to overcome these shortcomings but are sensitive to domain shift. This work shows that transfer learning (TL) combined with an ensembling strategy can help alleviate this key challenge. We evaluate four approaches: pre-trained models from the source domain, models directly trained from scratch on target domain data, models fine-tuned from a pre-trained model and an ensemble of fine-tuned models. For that the data base was split into 16 source and 4 target domain subjects. Comparing ensemble of fine-tuned models (N = 10) with directly learned models, we report significant improvements (P < 0.001) of the root mean squared error (RMSE) of up to 12% and the mean displacement (MDISP) of up to 17.5%. The smaller the target domain data amount, the larger the effect. This shows that TL + Ens significantly reduces beforehand acquisition time and improves reconstruction quality, rendering it a key component in making 4D MRI clinically feasible for the first time in the context of 4D organ motion models of the liver and beyond.

## Introduction

Insufficient compensation for irregular organ motion during image-guided interventions is a significant problem that can lead to inaccuracies in instrument navigation and compromised treatment outcomes. Real-time 4D MRI imaging in MRI-guided procedures holds the potential to address this issue. However, acquiring real-time 4D MRIs of a large target region during an intervention is currently not feasible due to the need for a significant amount of reference data beforehand. Although a recent study demonstrated promising results using a deep learning (DL) approach with only 24 min of training data^1^, this timeframe is still impractical for routine clinical settings where time is crucial. Additionally, there are limits to the specific absorption rate (SAR) allowed during MRI imaging, and these limits are likely to be exceeded during prolonged imaging. Consequently, the effective application of 4D MRI in the intervention room remains challenging. That could soon change with further advances in deep learning, as we will show in our work.

**Related work** 4D MRI methods can be classified as either respiratory phase-resolved or time-resolved (see Table 1). The former can reconstruct a fixed number of phases of a single breathing cycle (usually 10 or fewer phases) and can not account for arbitrary/irregular breathing. They are mainly based on sequence programming and unique k-space sampling designs, and the acquisition usually takes around 5 min. Cai et al.^2^ retrospectively sort axial slices into respiratory phases using the body area as an image-based internal respiratory surrogate. Hu et al.^3^ use single-shot acquisition with parallel imaging and partial k-space imaging to improve acquisition speed. They reconstruct four respiratory states of one breathing cycle. Tryggestad et al.^4^ acquire sagittal or coronal slices and retrospectively stack them in a two-pass approach into ten respiratory phase volumes. Paganelli et al.^5^ removed the need for navigator frames by directly comparing neighboring slices using mutual information to reconstruct one breathing cycle. Deng et al.^6^ implemented a continuous spoiled gradient echo sequence with 3D radial trajectory and 1D self-gating for respiratory motion detection to retrospectively sort data into different respiratory phases. Han et al.^7^ repeatedly sample the k-space center line as a self-gated motion surrogate and retrospectively bin k-space data into different respiratory phases. Lind et al.^8^ acquire coronal slices and extract an image-based self-sorting signal performing rigid registration of the diaphragm to sort the image data into ten respiratory phases retrospectively. Meschini et al.^9^ cluster data slices without using navigator slices by comparing different surrogate signals. Yang2020 et al.^10^ use the diaphragm in sagittal slices as an anatomic feature to sort axial slices into ten breathing phases. Eldeniz et al.^11^ train a deep learning network without ground truth to remove reconstruction artifacts from under-sampled phase-resolved 4D MRI.

4D MRI methods of the other class can reconstruct arbitrary/irregular breathing and are mainly based on clinically available MRI sequences. On the downside, most of these methods have long beforehand acquisition times of up to 60  min and are not real-time capable due to long reconstruction times of tens of seconds^12^. In 2007, Siebenthal et al.^13^ proposed a 4D MRI reconstruction framework for liver MRI with arbitrary breathing motion. They acquired a series of 2D MRIs alternating between spatially fixed navigators and spatially moving data slices. Using a search strategy, these multiple dynamic MRIs were then used to reconstruct corresponding 3D MRIs for any reference navigator. That way, they reconstructed time-resolved 4D MRI from dynamic 2D navigator sequences, which could be used as a precursor for a motion model. The shortcoming of this method is the long acquisition time needed to establish the data set in which the search is performed and the time-expensive search during reconstruction itself. Several works adapted the idea of Siebenthal and tried to address the long acquisition time within the framework. They applied machine-learning methods to interpolate navigators or data slices, effectively reducing acquisition time. From these approaches, the one of Tanner et al.^14^ is most similar to ours because it is based on learning the relation between navigator and data slices. The main difference is that their method is not an end-to-end learnable formulation. It requires a time-expensive search for similar data slices within the prior acquisitions, making the method suited for retrospective reconstruction only. Karani et al.^15^ train a convolutional neural network to temporally interpolate navigators and use that to effectively half the number of navigator acquisitions. Zhang et al.^16^ expanded on that idea and proposed temporal interpolation using the prediction of a motion field as an intermediate step reducing the problem of blurry predictions and missing structures. Yuan et al.^17^ proposed a time-resolved large FOV 4D MRI reconstruction technique. It is based on sequence programming to shorten MRI acquisition times drastically. It attains high temporal ($615\, \textrm{ms}$) at moderate spatial resolution ($2.7\times 2.7\times 4.0\, \textrm{mm}^{3}$). However, it is not real-time capable because the volume reconstruction takes around ${20}\, {\hbox {s}}$. Also, the huge amount of captured data (91 MR images/s) risks filling up the scanner’s memory during longer imaging sessions. Gulamhussene et al.^12^ improved reconstruction speed and robustness against the out-of-plane motion in the navigator by applying template updates.

All methods from both groups mentioned above reconstruct 4D MRI retrospectively. They can not reconstruct prospectively or in real-time, not to be confused with prospective and retrospective gating. In 2022, we proposed a novel near-real-time, time-resolved 4D MRI framework^1^. It is an end-to-end DL-formulation and based on the same acquisition scheme proposed by Siebenthal et al.^13^ but removes the active search for data slices by learning the relation between navigator and data slices and by that speeds the reconstruction up to sub-seconds ($\le 600 \textrm{ms}$). Unlike most related work, it performs extrapolation instead of interpolation and can thus be used in real-time during an intervention. It yields a large FOV, high temporal resolution, and a high isotropic spatial resolution of $({1.8}\,\text {mm})^3$. Still, our approach required half an hour of beforehand acquisitions for training. In essence, using previous works, one had to choose between long acquisition times and limited breathing phase support, i.e., no irregular breathing, none of which is clinically acceptable.

### Contribution

In this work, we solve the shortcoming of our previously proposed methods’ long acquisition time for predicted time-resolved 4D MRI^1^. First, we identify domain shift as a major issue for DL-based 4D MRI prediction, which gets more severe the smaller the amount of available target domain data is, which fits into the observations of a recent 2021 survey of Guan et al.^18^. Second, we show that the beforehand acquisition time can be substantially reduced (from 24  to 2 min) by using transfer learning (TL) techniques without losing the support for irregular breathing. Third, by combining multiple models in an ensemble strategy, we are able to mitigate the negative impact of reduced training data and improve the accuracy and reliability of the predictions.

**Table 1:** Comparison with the related work regarding whether reconstruction is done pro-/retrospectively (P/R), matrix size, voxel resolution, whether its time-resolved (TR), how many phases of a breathing cycle can be resolved (breath. cycle sampling), volumes per second (vps) in pro- and retrospective reconstruction (P/R), beforehand acquisition time (befAcq), reconstruction time, and RMSE and MDISP.

	Year	P/R	Matrix size	Resolution in mm^3^	TR	Breath. cycle smpl.*		fps		befAcq in min	Recon. time in s/vol.	RMSE mean (95%)	MDISP mean (95%)
						P	R	P	R				
Cai	2011	R	256 × 166	1.5 × 1.5 × 5	No	–	4	–	–	–	–	–	–
Hu	2013	R	250 × 176 × 32	1.5 × 1.5 × 5	No	–	4	–	–	3	–	–	–
Tryggestrad	2013	R	175 × 190 × 9	2 × 2 × 5	No	–	10	–	–	13	–	–	–
Paganelli	2015	R	256 × 224 × 20	1.28 × 1.28 × 5	No	–	8	–	–	1.2	–	–	–
Deng	2016	R	–	–	No	–	10	–	–	8	–	–	–
Han	2017	R	416 × 250 × 125	**1.2 × 1.2 × 1.6**	No	–	8	–	–	5	75	–	–
Lindt	2018	R	138 × 208 × 30	2 × 2 × 5	No	–	10	–	–	5	30	–	–
Meschini	2019	R	256 × 224 × 20	1.28 × 1.28 × 5	No	–	8	–	–	1.2	262	–	–
Yang	2020	R	–	1.67 × 1.67 × 5	No	–	10	–	–	–	–	–	–
Eldeniz	2021	R	318 × 318 × 96	1.13 × 1.13 × 3	No	–	10	–	–	5	2.7	–	–
Siebenthal	2007	R	192 × 192 × 25	1.8 × 1.8 × 4	**Yes**	–	**36**	–	5	60	73	–	0.68 (1.63)
Tanner	2014	R	224 × 224 × 53	1.3 × 1.3 × 5	**Yes**	–	**36**	–	4.4	10	–	–	0.8 (1.57)
Zhang	2018	R	–	1.33 × 1.33 × 5	**Yes**	–	**36**	–	2.4	30	36.5	10.23 (13.74)	0.36 (–)
Karani	2018	R	–	1.33 × 1.33 × 5	**Yes**	–	**36**	–	2.4	20	–	4.09 (6.81)	0.92 (2.62)
Yuan	2019	R	128 × 128 × 56	2.7 × 2.7 × 4	**Yes**	–	9.78	–	1.63	**0.33**	20	–	–
Gulamhussene	2020	R	140 × 176 × 47	1.82 × 1.82 × 4	**Yes**	–	**36**	–	**6**	60	27	–	–
Gulamhussene	2022	**P/R**	128 × 128 × 209	1.8 × 1.8 × 1.8	**Yes**	**10.5**	**36**	**1.75**	**6**	24	**0.57**	**0.24 (0.37)**	**0.35 (0.81)**
Our direct	–	**P/R**	128 × 128 × 209	1.8 × 1.8 × 1.8	**Yes**	**10.5**	**36**	**1.75**	**6**	2	**0.57**	0.34 (0.49)	1.83 (3.29)
Our TL	–	**P/R**	128 × 128 × 209	1.8 × 1.8 × 1.8	**Yes**	**10.5**	**36**	**1.75**	**6**	2	**0.57**	0.31 (0.48)	1.61 (3.11)
**Our TL + Ens (N = 10)**	–	**P/R**	128 × 128 × 209	1.8 × 1.8 × 1.8	**yes**	**10.5**	**36**	**1.75**	**6**	2	**0.57**	0.3 (0.46)	1.51 (2.98)

Values are taken from respective publications. Blank cells indicate information that was not reported in the respective work. Best values are bold. Our method with TL represents the best trade-off between befAcq time, prediction quality, FOV, and reconstruction time. *Based on a 6 s breathing cycle.

## Materials and methods

### Data acquisition

**Figure 1:**
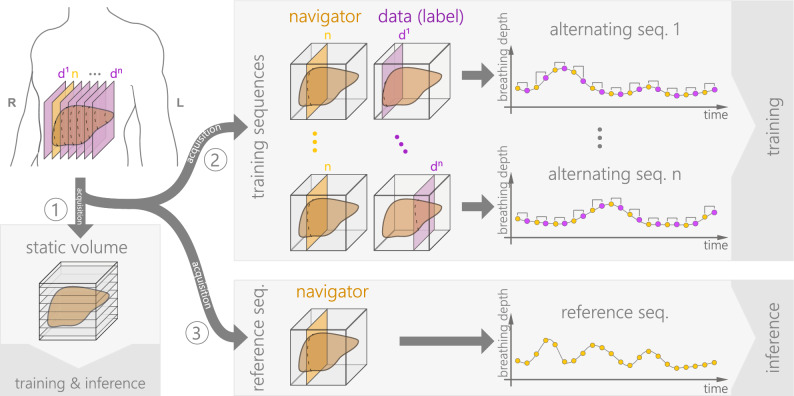
For each subject, three kinds of data were acquired. (1) A static volume, (2) several alternating dynamic sequences (brackets indicate pairs of navigators and data slices), and (3) a dynamic reference sequence. Figure content is based on a previous conceptual sketch (Fig. 1 in Gulamhussene et al.^1^).

The liver MRI data of 20 healthy subjects were acquired on a MAGNETOM Skyra MRI scanner (Siemens Medical Solutions, Erlangen, Germany), following the protocol described in our earlier work^1^. The data, study information, and MR sequence protocols are publicly available^19,20^. For each subject, the data comprises three parts (see gray boxes in Fig. 1), described in the following:

#### Static volume

The static 3D liver volume is used as an anatomical reference during training and inference. It is acquired with a STAR VIBE MR Sequence (320 $\times$ 320 $\times$ 72–88 matrix size, 3 mm slice thickness, $1.19 \times {1.19}\, \text {mm}^2$ in-plane resolution, 0% phase oversampling, 44.4% slice oversampling, 380 mm FOV read, 100% FOV phase, 2.83 ms TR, 1.48 ms TE, ${9}^\circ$ flip angle, 7/8 slice partial Fourier).

#### Training sequences

During free breathing, several dynamic 2D sequences were acquired, in which navigator slices alternate with data slices. Navigators and data slices form pairs and are used as training samples. As the name suggests, training sequences are used only during training. While the navigator slice position is fixed in the right liver lobe, the data slice position is unique for all sequences, equidistantly sampling the liver from right to left. The navigator shows several vessel cross-sections and serves as a respiratory motion signal. Each training sequence consists of 175 navigators and 175 data slices. For each subject, the number of training sequences ranged between 38 and 57, depending on the size of the subjects’ livers. Overall the acquisition time ranged between 40 and 80 min per subject.

#### Reference sequence

Also, during free breathing, a dynamic 2D sequence of navigator slices was acquired as a reference sequence. It is dynamic in time but static in position, i.e., the navigator has the same fixed position as in the training sequences. The reference sequence contains a natural succession of different breathing cycles/patterns, like shallow/deep and thoracic/abdominal breathing. It is used for inference as a respiratory reference, i.e., a breathing signal. The reference sequence comprises 513 time points in our data, covering 85 seconds (typically about 20 breathing cycles).

Both, training as well as reference sequence were acquired using a TRUFI MR sequence (39.96 ms TR, 3.33 ms echo spacing, 1.49 ms TE, ${30}^\circ$ flip angle, 676 Hz/voxel readout bandwidth, 176 $k_x$ base resolution, 80% phase resolution, 14$\times$176 matrix size, $1.8\times {1.8}\,\text {mm}^2$ in-plane resolution, 4 mm out of plane resolution, $255\times {320}\,\textrm{mm}^2$ FOV). For faster measurement, a partial Fourier was used, sampling 5/8 of the k-space asymmetrically in phase-encoding direction, i.e., roughly 60% of the $k_y$ lines, resulting in 88 acquired lines. This resulted in an acquisition time of 166 ms/slice. No body array coil was used.

The ethics board of the Otto-von-Guericke-University Magdeburg/Germany approves our study ”Studies with healthy subjects in 3 Tesla for methodological development of MRI experiments” (approval number 172/12), concluding that there are no ethical concerns. All research was performed in accordance with relevant guidelines and regulations. Verbal and written informed consent was obtained from all subjects.

**Figure 2:**
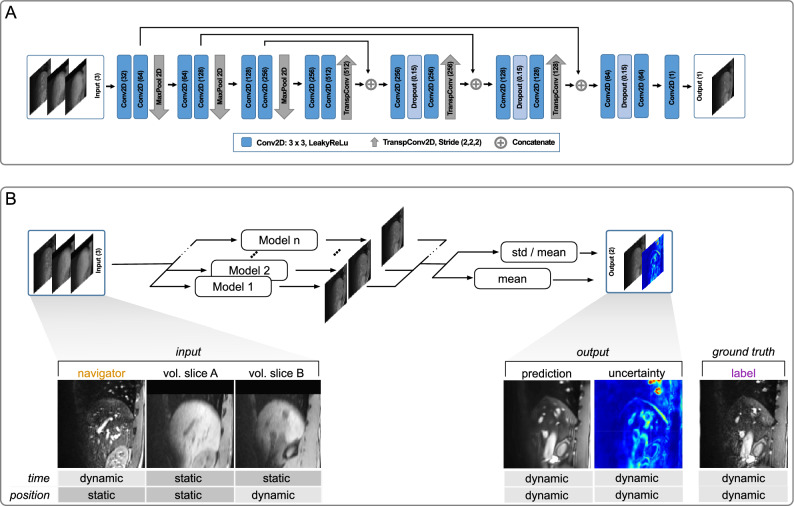
(**A**) U-Net architecture, with three-channel input. Blue boxes are convolutions; grey arrows are max pooling or upsampling, and pluses are feature map concatenations. (**B**) Ensembling of n models and generation of uncertainty map.

### Deep learning prediction of 4D MRI

(All source code will be publicly available upon publication).

#### Deep learning formulation

A deep network with three 2D input channels is trained using training sequences together with slices of the static volume. Each training input corresponds to a specific subject. However, samples from different subjects can be used. A training input consists of three channels (see Fig. 2). Pairs of navigator and data slices are taken from the training sequences of a subject. The navigator is fed to the first channel. The data slice serves as ground truth (label). Two slices are sampled from the static volume (from the same subject): one slice at the navigator position is fed to the second channel, and one at the ground truth position (the slice to be predicted) is fed to the third and last channel.

In the following, we explain the rationale of the three channels. The navigator (first channel) is dynamic in time and static in its position. It determines (shows) the breathing state. The volume slice at the navigator position (second channel) is static in both time and position and acts as a still reference to the dynamic navigator. It contains information on the relationship between the two different MR contrasts of the TRUFI and STAR VIBE MR images. The volume slice at the label position (third channel) is static in time but dynamic in position and acts as a still reference to the dynamic output we seek to predict and expresses the position that should be predicted.

During inference, the first channel determines the breathing state of the slice that is to be predicted, and the third channel determines its position. Thus we reconstruct any current breathing state (time domain) by providing a real-time navigator (acquired during the intervention) at any position (space domain) by choosing the proper position from the static volume (acquired before the intervention). By simply inferring all positions in one batch (in one forward pass), a total 3D volume for a time point is reconstructed. On a GeForce GTX 1080, this takes $\le {600}$ ms, yielding real-time 4D MRI. Furthermore, if reconstruction is done retrospectively—and graphics card memory permits—a whole 2D$+$T series at a fixed slice position or even a full 4D reconstruction can be performed in one batch.

#### Network architecture and training

In this work, we do not propose a new architecture. We use a U-Net^21^ to demonstrate our deep learning formulation. However, other architectures can be used as well. The only requirement is to have the three input channels described before. The three-channel input is processed in standard U-Net encoding and decoding paths. A leaky rectified linear unit (slope = 0.1) follows each convolutional layer. The convolutions are padded to keep the size of feature maps and input constant. The second convolutional layer in each block doubles the number of features, increasing the network’s capacity. A MaxPooling operation follows the first three blocks. In the first convolutional layer, 32 filters process the 128 $\times$ 128 $\times$ 3 input to the network. Following the architecture results in 512 feature maps in the latent feature space. The decoding reconstructs the image from the latent space. To this end, three blocks of two transposed convolutional layers are employed that up-sample the features. Between every two transposed convolutional layers, a dropout layer is used. With each up-sampling, the filter size is halved. A final $1\times 1$ convolutional layer outputs the reconstructed MR image.

The network was implemented with Keras^22^. The total of 6.8 million parameters are trained by an Adam optimizer^23^ (learning rate = 0.0004). We trained for 200 epochs using mean squared error (MSE) as loss and a batch size of 64. Checkpoints were employed and the model with the best validation loss was used. We applied z-score normalization, also known as whitening, to the image intensities of each subject. This normalization process ensured that the intensities had a zero mean and unit variance. It is important to note that this normalization was reversed after the prediction stage and specifically before the uncertainty map generation processes in the case of ensembling. The training data was augmented in a physiologically plausible range as described in our earlier work^1^ to facilitate robustness. Random augmentation was seeded for reproducibility. To simplify the processing, all images were re-sampled to ${1.8}\,\textrm{mm}^3$ voxels.

#### Transfer learning

As we will show in the next section, domain shift is a problem in MRI liver data and results in a discrepancy in model performance. We address this issue by fine-tuning a pre-trained model to a new target subject, because fine-tuning is a simple to use and effective technique. Its practicality and effectiveness make it particularly advantageous in a clinical context. Let **S** be the source domain and ${\textbf {s}} \in {\textbf {S}}$ be the subjects of the source domain. Likewise let **T** be the target domain and ${\textbf {t}} \in {\textbf {T}}$ be the subjects of the target domain. We use transfer learning in the form of fine-tuning to reduce the discrepancy in model performance in **S** and **T**. Specifically, let ${\textbf {M}}^{j}_{pre}$ be a pre-trained model that was trained on data from all N source domain subjects $[{\textbf {s}}_1, {\textbf {s}}_{N}] \in {\textbf {S}}$, where *j* denotes the minutes of training data per subject **s**. ${\textbf {M}}^{j}_{pre}$ is then fine-tuned with *i* minutes of training samples from a new subject ${\textbf {t}} \in {\textbf {T}}$ using the same training parameters as were used for the training of the pre-trained model (200 epochs, MSE loss, 64 batch size, data augmentation), resulting in the fine-tuned model ${\textbf {M}}^{i}_{pre+TL}$.

#### Ensembling and uncertainty map

We propose to combine an ensembling strategy together mit a transfer learning strategy with our 4D MRI framework. This is illustrated in Fig.2B). While fine-tuning does enhance prediction quality, when limited training samples are available, it may not completely mitigate the decrease in prediction quality caused by the smaller training data set. Ensembling plays an important role in addressing this issue. By combining multiple models, ensembling significantly improves the overall prediction quality and helps to mitigate the negative impact of the reduced training data set. To employ the ensembling strategy, N models were pre-trained, each starting from a random parameter initialisation. These N models were fine-tuned to a new subject following the training as described before. To form the final 4D MRI the predictions of the individual models in the ensemble are averaged. An uncertainty map is generated by computing the Coefficient of variation between the predictions. For that, after the normalization was reversed, the voxel wise standard deviation is dividing by the voxel wise mean.

## Experiments and results

We divided the 20 subjects into a source domain **S**, containing 16 subjects, and a target domain **T**, containing 4 Subjects. In both **S** and **T**, we used the first half of each training sequence as training data and the second half as validation data.

### Model performance

To quantitatively assess model performance and for statistical analysis, we use the following three image-based error measures that express the similarity of predicted MR slice and ground truth.

#### RMSE

We compute the RMSE of two images, i.e., predicted slice and ground truth, as expressed in Eq. (1) by computing the voxel-wise intensity difference $d_i$, then taking the root of the mean of the squared differences.

1 $$\begin{aligned} \text {RMSE} = \sqrt{\frac{1}{W \cdot H} \sum _{ i=0 }^{ W \cdot H } d_i^2 }, \end{aligned}$$

where **W** and **H** are the width and height of the images. It is common practice to report the RMSE in the evaluation of 4D MRI methods. However, the comparability of the measure across works is limited because different image normalization might be used. Moreover, this similarity measure does not differentiate between the appearance or presence of structures and the displacements of structures.

#### MDISP

We compute the MDISP by first performing a B-spline deformable registration using simpleITK^24^ to obtain a dense deformation field between prediction and label. The resulting dense deformation field was then sampled with a $16\times 16$ grid ($8\times 8$ voxel spacing) within the liver to obtain displacement vectors. We then compute the average Euclidean norm of the displacement vectors in mm. We manually segmented all livers in the static volumes and used the segmentation as a mask to sample only within the liver. The parameterization of the deformable registration algorithm was empirically determined as follows.

ANTSNeighborghoodCorrelation (radius $=$ 2) was used as the similarity measure. It visually yielded better registrations than MeanSquarse, MattesMutualInformation, and correlation. A pyramid scheme with two levels was utilized. In the first level, the images were smoothed with a sigma of 0.25 before halving their resolution using linear interpolation. In the second level, the original image was used with no smoothing. The grid size of the deformation mesh was $4\times 4$ in the first level. It was doubled to $8\times 8$ in the second level. A gradient descent optimizer (learning rate $=$ 0.25, number of iterations $=$ 20, convergence minimum value $=$ $1e^{-7}$, convergence window size $=$ 10, estimate learning rate = True, maximum step size in physical units $=$ 0.25) was used.

The MDISP is a better measure for comparison across works than the RMSE because the displacement of structures is independent of image normalization. However, the displacement field between a generated image and the ground truth is not always well defined. For example, when the prediction contains structures not present in the label or vice versa when structures are missing. An extreme example is an empty prediction, which would lead to an MDISP of zero, which of course, would not reflect the actual similarity.

#### DN_RMSE

To alleviate some of the shortcomings of RMSE and MDISP, we propose a new measure: the deformation-normalized root mean squared error (DN_RMSE). It computes the RMSE after the prediction is deformably registered to the label. Thus DN_RMSE measures the similarity purely based on appearance and not deformation or displacement and can be used to interpret small MDISP values better. Like MDISP, taken by itself, DN_RMSE is not conclusive. However, combined with MDISP, it aids in a better comparison of generated images within one work.

**Figure 3:**
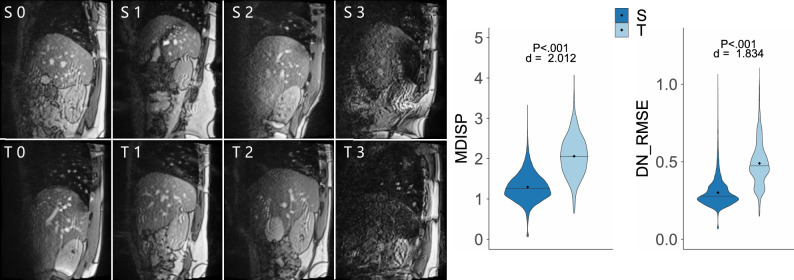
Navigators show considerable variance in anatomy and SNR , as illustrated by four source domain subjects (S0 to S3 ) and four target domain subjects (T0 to T3 ). The violin plot (right) shows the prediction error of a pre-trained model in the source domain (S) and the target domain (T).

### Domain shift

In this study the term domain shift is used in a general way, where it refers to the situation that the data distribution in the training set is different from the test set. And that this leads to a decrease in model performance. We argue that in clinical settings, the quantity of available training data is limited, and that there is a high likelihood that a new subject may not be adequately represented by the training set distribution. The inadequate representation of the new subject by the training set can be considered as domain shift. In our case, a small training distribution does not faithfully represent the following variations: liver shape and size, body height, abdominal girth and, consequently, SNR ( signal-to-noise ratio), body fat, sex, and age. This list might not be exhaustive. A tabular comparison of these aspects between the source domain and target domain can be found as Supplementary Table S1. To ensure anonymity only min, max, and mean values are reported. The liver shape is approximated as the extend along the three orthogonal directions SI (superior, inferior), AP (anterior, posterior), and LR (left, right). One can see that most values have a wide range between minimum and maximum. For example, the body height ranges from 160  to 220 cm , the body weight from 54  to 112 kg , and the liver volume from ${1182}\,$ to ${2435}\,\textrm{cm}^3$ . Also the liver extent has wide ranges in all three orientations (SI, AP, LR). A comparison of the different liver shapes and apparent SNR between source and target domain is given in Fig. 3. It is likely that the 16 source subjects do not represent the distribution of all factors over these wide ranges faithfully .

Remember, ${\textbf {M}}^{24}_{pre}$ is a model pre-trained on all 16 Subjects from the source domain **S**, using ${24}\,\textrm{min}$ worth of training samples per subject. Of course, it would be best if it could be applied to a new subject ${\textbf {t}} \in {\textbf {T}}$ directly and without any adaptation. However, this requires that there is no domain shift present between **S** and **T**. To test this, we compare the domains in two ways. First, the performance of ${\textbf {M}}^{24}_{pre}$ is compared between validation data (from **S**) and test data (**T**) using the MDISP and DN_RMSE. To that end, we randomly chose 50% of test samples from the first 10 seconds of the second half of each training sequence, i.e., for each subject (in **S** and **T**) and slice position. We then computed both similarity measures for all predictions of the test samples. Second, the anatomical variance was assessed visually using the navigator frames. We visualize the MDISP and DN_RMSE distributions in a violin plot (see Fig. 3). The violin plots show non-normal distributions with different mean. Because a Shapiro-Wilk Test (n $=$ 4000) and Kolmogorov-Smirnov test also showed that the distributions are not normally distributed (p < 0.001), we used a Wilcoxon rank sum test (m $=$ 3040, n $=$ 12,352) to test for significance of the distribution shift. The null hypothesis of no shift in error distribution was rejected at a significance level of p < 0.001. The mean of MDISP and DN_RMSE are 0.30 and 1.29 in **S** and 0.49 and 2.06 in **T**. We quantify the effect size with Cohen’s d (n $=$ 3040, m $=$ 12,352) and find the effect size is large with d $=$ 2.01 and 1.834. The visual comparison of the navigators shows variability in liver anatomy across subjects concerning the superior–inferior extent of the liver and the number and arrangement of vessels. This leads us to believe that domain shift is the reason for the significant shift in performance outcome of ${\textbf {M}}^{24}_{pre}$ in **S** and **T**.

**Figure 4:**
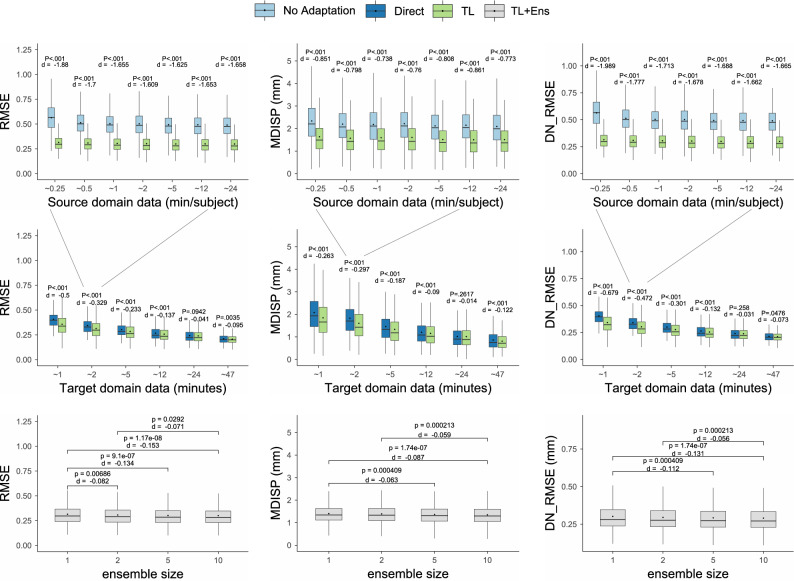
Top: comparison of no adaptation and TL at different levels of source domain data. Middle: comparison of direct learning and TL at different levels of target domain data. Bottom: comparison of ensemble sizes.

**Figure 5:**
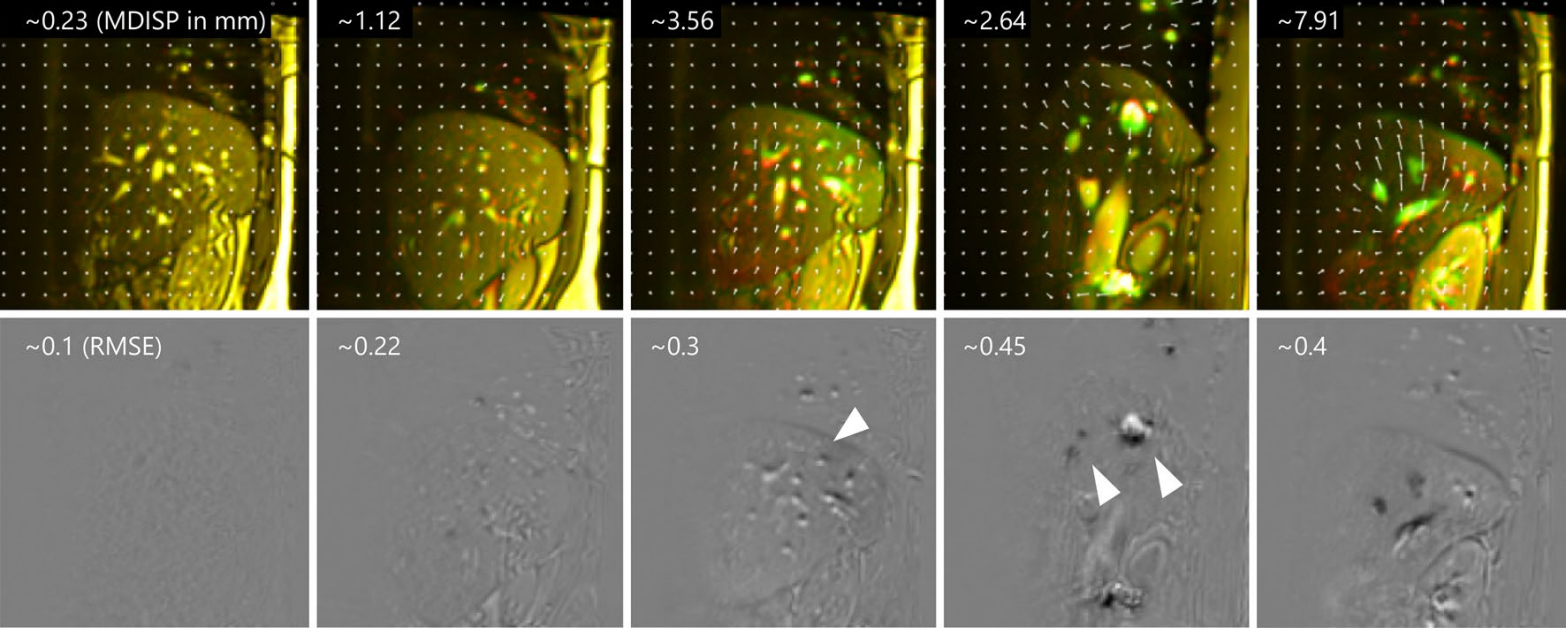
Top row: displacement fields with a composite of (red) labels and (green) predictions as reference. Bottom row: intensity differences images.

**Table 2:** Comparison of our method with no adaptation (no A) and with TL and different availability of source domain data.

	Mean													
	15 s		30 s		1 min		2 min		5 min		12 min		24 min	
	No A	TL	No A	TL	No A	TL	No A	TL	No A	TL	No A	TL	No A	TL
RMSE	0.57	0.33	0.52	0.32	0.51	0.32	0.51	0.31	0.5	0.31	0.5	0.31	0.5	0.3
MDISP	2.65	1.64	2.21	1.57	2.19	1.59	2.22	1.61	2.12	1.51	2.15	1.5	2.1	1.51
DN_RMSE	0.56	0.31	0.51	0.31	0.5	0.3	0.5	0.3	0.49	0.3	0.49	0.3	0.49	0.3
	95th percentile													
RMSE	0.83	0.89	0.77	0.48	0.76	0.48	0.76	0.48	0.75	0.48	0.75	0.47	0.74	0.46
MDISP	4.42	2.98	3.87	2.93	3.77	3	3.82	3.11	3.65	2.81	3.65	2.8	3.63	2.79
DN_RMSE	0.82	0.46	0.76	0.45	0.76	0.45	0.76	0.46	0.75	0.45	0.75	0.45	0.74	0.45

### Pre-trained vs. TL and influence of source domain data availability

Because domain shift is a challenge in deep learning-based 4D MRI prediction, we propose to employ TL. We evaluate the effect of TL on our models by comparing ${\textbf {M}}^{j}_{pre}$ ($j \in [2, 5, 12, 24]$) with ${\textbf {M}}^{2}_{pre+TL}$ regarding their performance in **T**. Where ${\textbf {M}}^{2}_{pre+TL}$ is the result of fine-tuning ${\textbf {M}}^{j}_{pre}$ with 2 minutes of samples from **T** (720 samples = ${2}\,\textrm{min}$ acquisition time). By that, we also analyze how the source data amount *j* influences the effect of TL. For comparison, we use RMSE, MDISP, and DN_RMSE. The top row of box plots in Fig. 4 shows the results of this experiment. Two observations can be made. First, transfer learning improves the model performance in the target domain for all tested measures. All tested measures show a high significance level of p<.001. Significance levels were computed using the Wilcoxon rank sum test (m $=$ 3040, n $=$ 12,352) after confirming none normal distributions using the Shapiro–Wilk test (n $=$ 3040) and Kolmogorov–Smirnov test. We observe high effect sizes with $|\text {d}| > 1.6$ for RMSE and DN_RMSE and medium effect sizes with $|\text {d}| > 0.7$ for MDISP. Second, the amount of source domain data (beyond $\sim {1}\,\textrm{min}/\text {subject}$) has little to no influence on the effect size. It also does not affect the performance of either ${\textbf {M}}^{j}_{pre}$ or ${\textbf {M}}^{2}_{pre+TL}$ in **T**. In table 2 we report means and 95th percentiles.

**Table 3:** Comparison of our method with direct learning and with TL.

	Mean											
	1 min		2 min		5 min		12 min		24 min		47 min	
	Direct	TL	Direct	TL	Direct	TL	Direct	TL	Direct	TL	Direct	TL
RMSE	0.41	0.36	0.34	0.31	0.3	0.28	0.27	0.26	0.24	0.24	0.21	0.2
MDISP	2.08	1.85	1.83	1.61	1.46	1.33	1.2	1.15	1.01	1.01	0.86	0.81
DN_RMSE	0.4	0.34	0.34	0.3	0.3	0.28	0.26	0.25	0.24	0.24	0.21	0.21
	95th percentile											
RMSE	0.59	055	0.49	0.48	0.44	0.44	0.4	0.4	0.38	0.37	0.29	0.28
MDISP	3.67	3.57	3.29	3.11	2.67	2.65	2.23	2.27	1.97	1.98	1.64	1.54
DN_RMSE	0.57	0.51	0.48	046	0.42	0.43	0.39	0.39	0.36	0.36	0.29	0.28

Availability of target domain data given in minutes.

### TL vs. direct learning and the influence of target domain data availability

**Figure 6:**
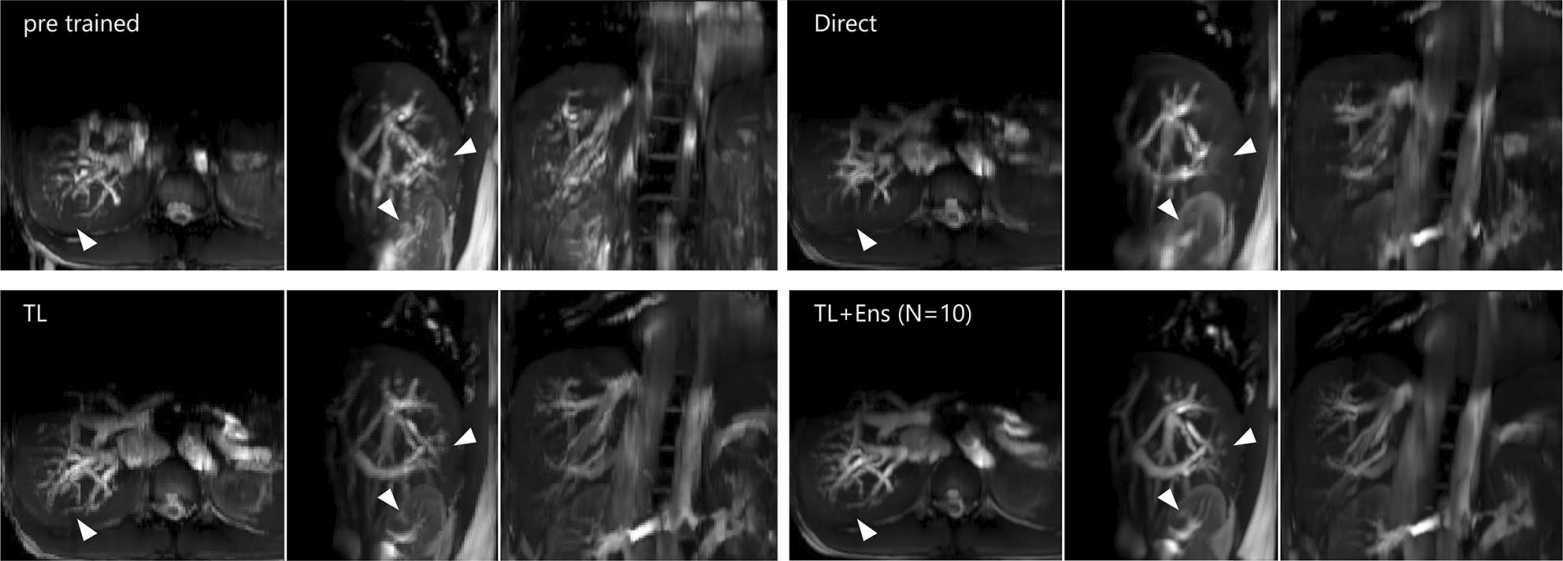
From top left to bottom right predictions of: ${\textbf {M}}^{2}_{pre}$, ${\textbf {M}}^{2}_{direct}$, ${\textbf {M}}^{2}_{pre+TL}$, ensemble of $10\times {\textbf {M}}^{2}_{pre+TL}$. Arrows indicate places of detail gain.

We evaluate whether TL is beneficial compared to directly learning a model from scratch in the target domain. Moreover, we evaluate how the target sample availability influences that effect regarding the effect size. To that end, we directly train models from scratch on samples from **T** and compare them with fine-tuned models. Let ${\textbf {M}}^{i}_{direct}$ be a directly learned model and let ${\textbf {M}}^{i}_{pre+TL}$ be a model fine-tuned from ${\textbf {M}}^{2}_{pre}$, where $i \in [1, 2, 5, 12, 24, 47]$. ${\textbf {M}}^{2}_{pre}$ was chosen as the base model because *j* showed virtually no influence on model performance in **T**. Furthermore, acquiring only a few samples to train a base model in a real-world scenario would be more economical. The model performance was tested dependent on the availability of target domain samples from 1 to 47 min (see the bottom row in Fig. 4). For each target data availability level *i* and target subject *t*, one model was trained directly and one with TL (in total, 48 models). For target data availability between 1 and 12 min, we observe significant improvements (p < 0.001) when using TL concerning RMSE, MDISP, and DN_RMSE, and visual assessment reveals detail gain (see Fig. 6). Beyond the level of ${12}\,\textrm{min}$, improvements are not significant. We find that effect sizes are largest (small to medium) between 1 and 12 minutes when few target samples are available and become negligible when large amounts of target samples are available. We used the Wilcoxon rank sum test (m $=$ 3040, n $=$ 3040) to test for significance after we checked that the distributions are not normally distributed using the Shapiro–Wilk test (n $=$ 3040) and Kolmogorov–Smirnov test. Effect sizes are reported as Cohen’s d. In Table 3 we report means and 95th percentiles. Figure 5 illustrates the image quality and displacement fields of predictions for increasing MDISP and RMSE values. We present 4D visualizations in this video: https://youtu.be/bh8A9SoAXvM. (The video’s visibility will be set to public once the manuscript is accepted. During review the video is provided as Supplementary Material).

### TL+Ens vs. TL

We evaluate whether the combination of transfer learning with the ensembling strategy (TL+Ens) enhances the model performance. For that, we compare ensembles of fine-tuned models of different ensemble sizes with regard to RMSE, MDISP, and DN_RMSE. Where the ensemble size N $=$ 1 represents only TL, i.e. no ensembling. A one-factorial analysis of variance (ANOVA) was performed to test for a primary effect of the ensemble size, which reveled a significant effect. A post-hoc pair-wise Tukey’s test was performed for the RMSE, MDISP, and DN_RMSE independently using p-adjustment. The pair-wise effect size was computed, using Cohen’s d. One can see that ensembles (TL + Ens) of size N = 5 and 10 perform significantly better than N = 1 (TL) in all tested metrics. Although ensembling provides some benefits, the effect size is relatively small, suggesting that our TL strategy has reached a saturation point in terms of quantitative result quality. However, based on a subjective perspective, our senior radiologists with extensive experience consistently preferred the results of the TL+Ens approach over the TL-only results in all tested cases. The boxplots and all pairwise significances and Cohen’s d are presented in Fig. 4. The mean and 95th percentile are reported in Table 4.

**Table 4:** Comparison of ensemble sizes N.

Mean (95th percentile)				
	N = 1	2	5	10
RMSE	0.31 (0.49)	0.31 (0.47)	0.3 (0.47)	0.3 (0.46)
MDISP	1.58 (2.98)	1.56 (3.04)	1.53 (3.04)	1.51 (2.98)
DN_RMSE	0.3 (0.46)	0.3 (0.45)	0.29 (0.44)	0.29 (0.44)

## Discussion and conclusion

The main advantage of utilizing TL and ensembling in our DL-based 4D MRI method is that it dramatically reduces the effect of domain shift. Moreover, the amount of target domain samples can be halved without hampering the model’s performance compared to direct learning. From a clinical perspective, TL makes our method more economical because less beforehand acquisition and, therefore, less patient time in the MRI machine is needed. This is where our method stands out the most from the related work (see Table 1). It enables short pre-imaging times while achieving high prediction quality concerning RMSE, MDISP, and DN_RMSE comparable with the related work. We evaluated our method with different amounts of training data for fine-tuning and believe 2 min yield a good balance between short acquisition time and high prediction quality. With 2 min, our method achieves a mean MDISP below voxel size with the 95th percentile below two voxels. Unlike the related work, our method is an extrapolation technique fast enough to predict real-time 4D MRI during an intervention, which is another unique strength. It should be noted that comparing the related work with our method regarding MDISP is a bit unfair because interpolation, where the used temporal context can extend into the future, is easier than extrapolation. Nonetheless, our method can also be used retrospectively and still be competitive.

It should be noted that although most tests showed high significance for our experiments, this is not the main point, especially where the effect size is small. In these cases, the high significance levels are caused by the large statistical sample size. Overall the effect size is of greater relevance. We have shown that the effect of TL is greatest when few training samples are available but becomes negligible for training sample sizes of 24 min and beyond. However, this matches with the clinical need for short acquisition times.

The data set used in this study contains only healthy subjects. New studies are needed to conclude how well the 4D MRI models generalize to patient data from image guided liver interventions and other clinical settings.

We chose fine-tuning as a simple yet effective way of transfer learning to exemplify the novel combination of transfer learning with the deep learning based 4D MRI method. Of course, more advanced techniques could help to gain additional quality, which should be investigated in the future.

At 2 min worth of training samples, our method requires a fraction of beforehand acquisitions compared to the related work but has a larger MDISP. It would be interesting to quantify the benefit of improving sub-millimeter precision in the context of medical imaging, where voxel sizes typically range from 1 mm to 2 mm and whether a mean displacement of $<1$ voxel might be sufficient. We see a few avenues to improve our method for future work. First, in the case of retrospective use, it would be interesting to increase the amount of training data by incorporating navigator interpolation^15,16^, and data interpolation^14^ to double the temporal resolution to 83 ms to increasing prediction quality. Second, it would be interesting to investigate the use of coordConv layers^25^ in place of normal convolutions to improve prediction quality. This seems very promising because the spatial component of the learning task is dominant. Lastly, a 3D architecture instead of a 2D one might make it easier to learn the 3D spatial relations of the liver motion. In that case, the training task could also be reformulated to directly predict the 3D motion field, which would be beneficial for use in radiation therapy.

We received positive feedback from two senior radiologists with extensive experience in image-guided liver interventions, who confirmed that the presented results would offer significant benefits if implemented in clinical practice. They preferred the TL+ensemble. Specifically, the translation of our work to the clinic could yield significant advantages in interventional planning and simulation. This would only be possible because of the very short pre-acquisition time. The significant reduction in pre-acquisition time is crucial for two reasons. Firstly, time is a critical clinical resource. Reducing the time required for pre-acquisition allows for more efficient and streamlined imaging procedures. Secondly, there are strict limits on the specific absorption rate (SAR), which measures the amount of energy absorbed by the patient during the MRI scan. Prolonged acquisition times could potentially exceed these limits and pose safety risks. Therefore, the ability to shorten the pre-acquisition time is not only advantageous for time management but also for ensuring compliance with SAR regulations. For future research, it would be intriguing to adapt our method to simulate the breathing motion of planning data from patients.

### Conclusion

In this work, we proposed to utilize TL and an ensembling strategy to substantially reduce beforehand acquisition time and improve the prediction quality of a DL-based 4D MRI prediction model. The approach uses only a few training samples for each new subject. Although demonstrated for the liver, it can be used for any organ affected by breathing motion. The method can be used in real-time for 4D imaging during image-guided interventions or retrospectively to create a 4D MRI as a precursor for a respiratory motion model for radiotherapy. We believe DL-based real-time 4D MRI with high spatial and temporal resolution has the potential to impact image-guided interventions and radiation therapy because it can help to solve the problem of organ motion without interfering with the clinical workflow.

## Supplementary Information

Supplementary Table S1.

Supplementary Video 1.

## Data Availability

The MRI data, study information, and MR sequence protocols used in this study are available in the Open Science Repository for Research Data and Publications of OVGU (Creative Common License 4.0) in part one: https://doi.org/10.24352/UB.OVGU-2019-093 and part two: https://doi.org/10.24352/UB.OVGU-2021-071. All source code will be publicly available via GitHub upon publication.
