# Supplementary material for: Transfer-learning is a key ingredient to fast deep learning-based 4D liver MRI reconstruction
Source: Sci Rep. 2023 Jul 11;13:11227. doi: 10.1038/s41598-023-38073-1 (PMC10336014; doi:10.1038/s41598-023-38073-1)
Supplement: Supplementary file 1 — Supplementary Table S1. [file 41598_2023_38073_MOESM1_ESM.pdf]

Supplementary material

Transfer-Learning is a Key Ingredient to Fast Deep Learning-Based 4D Liver MRI Reconstruction

		Source Domain (3 Women, 13 Men)			Target Domain (4 Men)		
		Min	Max	Mean	Min	Max	Mean
Body Height (cm)		160	220	182	174	197	184
Body Weight (kg)		54	112	82	80	88	84
Age (Years)		23	40	29	30	34	32
Liver Slice Count		38	61	48	41	52	48
Liver Volume cm^3		1182	2435	1736	1532	2013	1793
Liver Extent (mm)	SI	151	215	179	141	212	182
	AP	140	206	163	143	182	162
	LR	147	259	186	176	223	197
Samples Count		6817	11485	8812	7891	8961	8338
Acq. Time (min)		38	61	48	41	52	47

Supplementary Table S1: Comparison of the source domain and target domain regarding demographic data, liver size, and other meta-data.
